# Evaluation of perlite, wood shavings and corncobs for bedding material in rats

**DOI:** 10.4102/jsava.v88i0.1492

**Published:** 2017-03-30

**Authors:** Fatih Yildirim, Betül A. Yildirim, Ahmet Yildiz, Kübra A. Kapakin Terim, Seyda Cengiz, Selçuk Özdemir

**Affiliations:** 1 Department of Animal Science, Atatürk University, Turkey; 2 Department of Biochemistry, Atatürk University, Turkey; 3 Department of Pathology, Atatürk University, Turkey; 4 Department of Microbiology, Atatürk University, Turkey

## Abstract

Bedding material, which is a significant part of rodent housing, affects the health and well-being of laboratory animals. The aim of this study was to evaluate perlite as a bedding material for rodents and to compare it with wood shavings, expanded perlite and corncobs. The animals used in this experiment were 48 male and 48 female Sprague-Dawley rats. The bedding materials collected from experimental groups were analysed microbiologically. Blood samples from rats were subjected to biochemical analysis for catalase, glutathione, glutathione peroxidase, malondialdehyde, superoxide and dismutase, and foot pad skins of rats were subjected to histopathological examination. Body weight was determined at the end of the 30-day period. Perlite as the only bedding material had no effect on body weight, and it resulted in less microbial activity compared with the wood shavings, expanded perlite and corncobs. However, using perlite alone had negative effects on the skin, the moisture percentage of bedding and stress parameters. A wood shavings-perlite combination gave better results than perlite alone and appropriate perlite and other bedding material mixtures may result in bedding materials conducive to animal health and welfare. The frequency of changing the bedding material should be limited to once weekly.

## Introduction

The type of bedding used is an important component of rodent housing and has as impact on the health and well-being of laboratory animals. Types of bedding available, each with their own unique characteristics, include wood shavings, paper, corncobs and chips. The quality of a bedding material is closely related to its moisture content and factors such as the microbial, traumatic and aeration effects of its components (Kraft 1980; Potgieter & Wilke 1993). The importance of bedding material has led to many recent studies (Anand Babu & Prasad 2014; Bind et al. 2013; Burn & Mason 2005, 2008; Burn et al. 2006; Ferrecchia, Jensen & Van Andel 2014; Krohn & Hansen 2008; Maxim, Niebo & McConnell 2014; Peace et al. 2001; Ras et al. 2002).

Wood shavings, which are composed of fine particles of wood, have recently been commonly preferred as the bedding material of choice for laboratory animals because of its low cost. However, it is now avoided because of toxicological concerns (Dean 1999).

Corncob bedding is obtained from granulated cobs after the corn kernels have been mechanically removed. It has become an increasingly popular selection for rat bedding. It is one of the hardest beddings available and contains no dust, reduces the spread of allergens and produces negligible levels of ammonia within the cage. Thus, it reduces the number of cage changes necessary (Krohn & Hansen 2008; Perkins & Lipman 1995; Ras et al. 2002). However, in spite of the advantages of corncob bedding, some scientists prefer other types of bedding because its resting surface is deemed to be uncomfortable for rats (Krohn & Hansen 2008; Ras et al. 2002).

Perlite is an amorphous volcanic glass that expands when rapidly heated at 760 °C – 980 °C (Maxim et al. 2014). It has primarily been used for building materials and as a horticultural aggregate (Ciullo 1996). The water absorbency of perlite is relatively high, and it poses no apparent health hazards (Berge 2009). Rodent bedding should be able to absorb moisture from urine and faeces, and perlite does this as well as reducing bacterial growth and production of gases, such as ammonia and carbon dioxide (Burn & Mason 2005; Hawkins et al. 2003).

Oxidative stress is defined as an imbalance between the production of free radicals and reactive metabolites (the so-called oxidants or reactive oxygen species [ROS]) and their elimination by protective mechanisms, referred to as antioxidants. This imbalance leads to damage of important biomolecules and cells, with a potential impact on the whole organism (Durackova 2010). The harmful effects of ROS are balanced by the action of antioxidants, some of which are enzymes present in the body (Halliwell 1996). Oxidative stress can be determined with catalase (CAT), glutathione peroxidase (GPx), malondialdehyde (MDA), superoxide dismutase (SOD) and glutathione (GSH) measurements (Sisein 2014).

This study aimed to evaluate perlite as a bedding material for male and female Sprague-Dawley rats and compare it with wood shavings, perlite and corncobs.

## Materials and methods

### Animals

In this study, 48 male and 48 female one-month-old Sprague-Dawley rats weighing 60 g – 70 g were used for the experiments. Their initial body weights were randomised in groups. The rats were obtained from the Medicinal and Experimental Application and Research Center (ATADEM), Erzurum, Turkey. Moreover, these rats were specific-pathogen-free and had conventional (undefined) gastrointestinal tract flora. They were maintained under a 12-h light-dark cycle (lights on at 07:00) in a temperature-controlled room (21 °C ± 2 °C) with a relative humidity of 50% – 70%. Water was given to animals via water bottles, and the animals were fed a normal diet in a wire insert. The animals were allowed free access to food and water during the experiments. All sample rats were collected individually for consistency. The body weight of each rat was recorded at the end of the study.

### Housing conditions

The rat cages were made of polycarbonate plastic and were 18 cm high, with an approximate 836 cm^2^ footprint. Rats were divided into 16 groups (six rats in each group) according to sex (male and female), bedding material (wood shavings, wood shavings-perlite, perlite and corncobs) and bedding-change frequency (once or twice per week).

### Bedding

The bedding materials were wood shavings (ATADEM, Erzurum, Turkey) approximately 1 cm – 2 cm long and 0.5 cm – 1 cm wide, expanded perlite (Kaleblokbims^®^, Erzurum, Turkey) and corncobs (^1^*/*_5_’’) (M.B.D. Food Company, Kocaeli, Turkey). One litre of bedding was placed into the rat cages. Bedding was changed once or twice per week.

### Moisture percentage of bedding

The percentage of moisture of each bedding material was determined according to dry matter using a drying oven. The samples were obtained from two areas 10 cm away from the water bottle and were then homogenised. The samples were randomly taken from each cage during bedding changes once a week at 7, 14, 21 and 28 days, and twice a week at 4, 7, 11, 14, 18, 21, 25 and 28 days. Drying was performed at 130 °C for 1.5 h. At least two determinations per sample were performed.

### Microbiological analysis of bedding

Samples for microbiological analysis were randomly taken from each cage during bedding changes once a week at 7, 14, 21 and 28 days. Samples of 10 g of each pooled bedding material were homogenised with 100 mL of sterile peptone water. The spread plate count method was used for bacterial enumeration (Maturin & Peeler 2001). The homogenised pooled bedding materials were subjected to serial dilution (dilution factor of 1/10) using a sterile saline (0.85% NaCl) diluent. A volume of 0.1 mL from each dilution was spread onto nutrient agar for the total bacterial count. Plates were incubated at 37 °C for 24 h.

### Blood analysis

The rats in each group were sacrificed by cervical dislocation after 30 days under sevoflurane (Sevorane liquid 100%, Abbott Laboratory, Istanbul, Turkey) anaesthesia, and blood was collected from the hearts for biochemical analysis. The collected blood samples were then transferred to vacuum tubes containing anticoagulant (lithium heparin), and plasma was separated by centrifuging at 3000 rpm at 4 °C for 10 min by Hettich^®^ ROTINA 38/38R 240V centrifuge and stored at -20 °C until the biochemical analysis was conducted. Plasma CAT activity was measured with difference in hydrogen peroxide at 240 nm according to the method of Goth (Goth 1991). GPx level of the plasma was measured at 412 nm according to the method of Matkovics (Matkovics, Szabo & Varga 1988). MDA level in the plasma was measured using the thiobarbituric acid reaction at 535 nm according to the method of Yoshioka et al. (1979). The superoxide radicals were produced by the xanthine and xanthine oxidase system, following the reaction of nitro blue tetrazolium and the formation of formazan dye, which was used to measure SOD activity according to the method of Sun, Oberley and Li (1988), and its activity was measured as the level of inhibition of absorbance at 560 nm (Sun et al. 1988). GSH level of the plasma was measured at 412 nm according to the method of Tietze (1969). The concentrations of the CAT, GPx, MDA, SOD and GSH were represented as kU/L, U/mL, mmol/L, U/mL and mmol/L, respectively, and were measured by Biotek microplate reader (Biotek μQuant MQX200 Elisa Reader, Winooski, VT, USA).

### Histopathological analysis

The foot pad skins of the rats in each group were removed and immediately fixed in 10% neutral buffered formalin. After dehydration in a graded ethanol series and cleaning with xylene, the sample material was embedded in paraffin, and 4-μm-thick sections were stained with haematoxylin-eosin (HE) for observation under a lighted microscope. Tissue sections were evaluated by a high-power light microscopic examination using an Olympus Bx51 with a DP72 camera (Olympus America Inc., Center Valley, PA, USA) system. Ten microscopic fields were randomly examined at 20x magnification. The scores of epithelial degeneration and inflammation were derived semi-quantitatively using light microscopy on the preparations from each rat and were reported as follows: none: − (0), mild: + (1), moderate: ++ (2), severe: +++ (3) and very severe: ++++ (4).

### Statistical analysis

Results are presented as means and the pooled standard error of the mean (SEM). Data were tested by a general linear model using IBM SPSS Statistics for Windows (Version 20, IBM Corp., Armonk, NY, USA). In this study, male and female rats were evaluated both separately and together. Differences were considered significant at *p* < 0.05. When an analysis revealed a significant difference, the differences among specific groups were then analysed using Tukey’s multiple comparison *post hoc* test (*p* < 0.05).

## Results

### Bedding material effects on body weight

**FIGURE 1:**
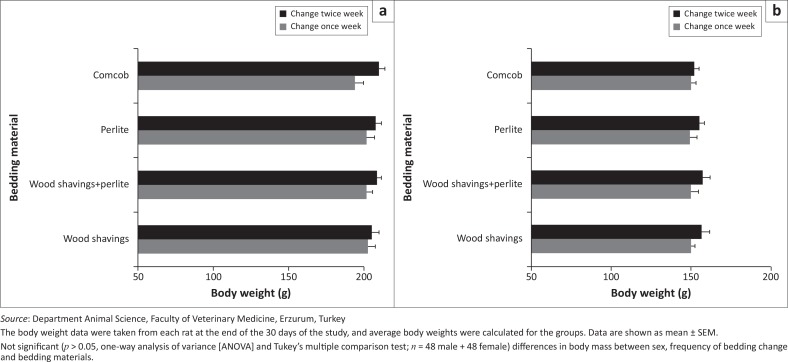
Body weight differences between groups according to sex ([a] male and [b] female), frequency of bedding change and bedding material.

Bedding material had no significant effect (*p* > 0.05) on body weight among the groups (wood shavings, wood shavings-perlite, perlite and corncobs). Although there were no significant differences among groups considering bedding-change frequency, the body weight was higher in rats whose bedding was changed twice than in those whose bedding was changed once per week (Figure 1). Male rats gained more weight than female rats by the end of the experiment (*p* < 0.001).

### Moisture percentage and bacterial count of bedding materials

**FIGURE 2:**
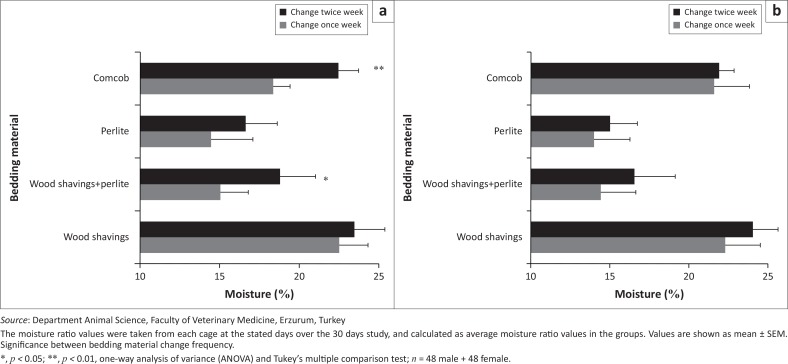
Moisture ratio (%) of bedding material with once (7, 14, 21 and 28 days) and twice (4, 7, 11, 14, 18, 21, 25 and 28 days) per week changes ([a] male and [b] female).

**FIGURE 3:**
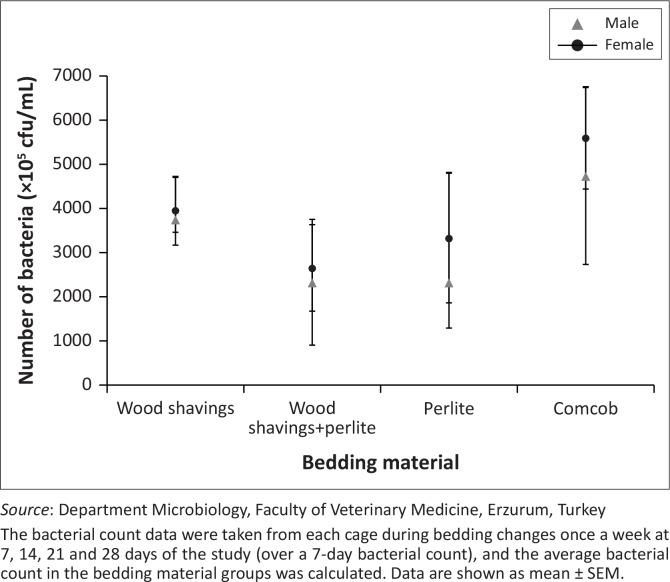
Bacterial counts in different types of bedding for male and female rats (*n* = 24 male + 24 female).

The moisture percentages of perlite and the wood shavings-perlite combination were shown to be lower than those of corncobs and wood shavings throughout the study. In male rats, bedding-change frequency had a significant effect (*p* < 0.05) on the moisture percentage of corncobs and wood shavings-perlite. Moisture percentage was higher in rats whose bedding was changed twice per week (Figure 2). Total bacterial count was higher in female rats than in male rats. Moreover, total bacterial counts for corncobs were higher than that of all the other bedding materials (Figure 3).

### Histopatological analysis of food pad skin of the rats

**FIGURE 4:**
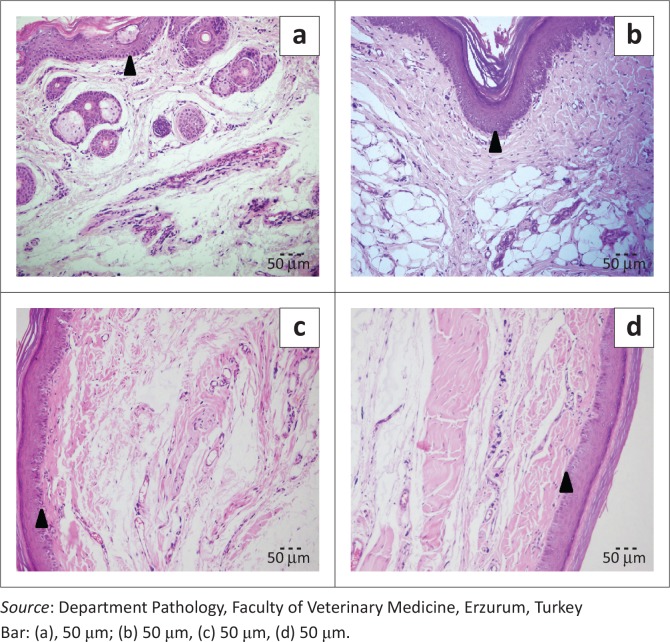
Bedding-change frequency (once per week): Degenerative changes (arrow head) in epithelial cells, H&E, (a) perlite (severe), (b) wood shavings-perlite (moderate), (c) wood shavings (mild) and (d) Corncob (mild).

**FIGURE 5:**
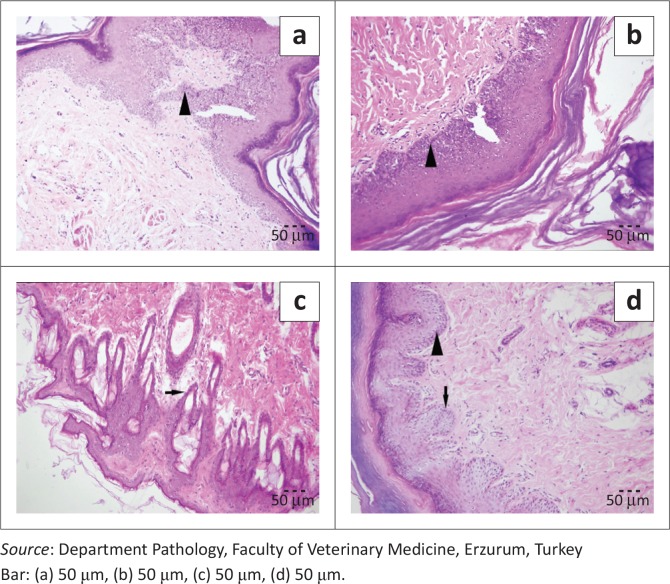
Bedding-change frequency (twice per week): Degenerative changes (arrow head) and acantholysis (arrow) in epithelial cells, H&E, (a) perlite, (severe), (b) wood shavings-perlite (moderate), (c) wood shavings (mild) and (d) corncob (mild).

**FIGURE 6:**
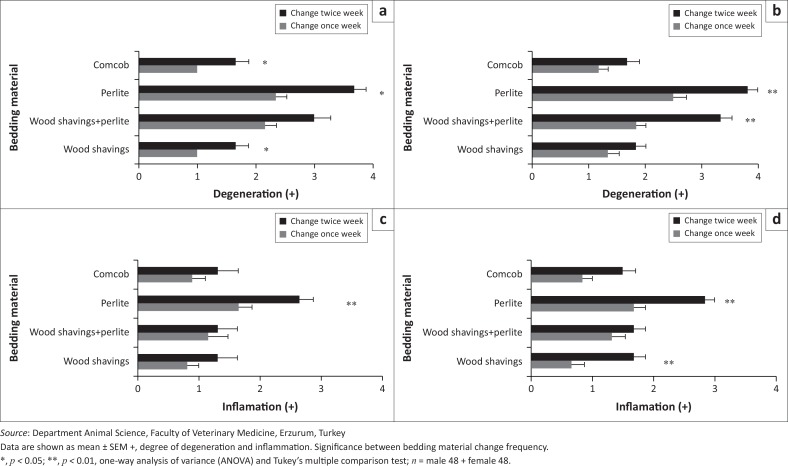
Histopathologic degeneration and inflammation degrees on the skin of male and female rats according to changing frequency and bedding material. (a) Male degeneration, (b) female degeneration, (c) male inflammation and (d) female inflammation.

Degeneration, acantholysis (Figures 4 and 5) and inflammatory reactions in epithelial cells of the skin were higher for rats with perlite bedding compared with those with other bedding materials (male: *p* < 0.01; female: *p* < 0.001). However, degenerative and inflammatory reactions on the skin were less with the wood shavings-perlite combination compared with perlite alone. In addition, changing the bedding twice per week increased degenerative and inflammatory lesions in all groups (male: *p* < 0.01 and female: *p* < 0.001) (Figure 6).

### Blood analysis of rats

The results of biochemical tests for CAT, GPx, MDA, SOD and GSH are shown in Table 1. In the perlite group, there was a significant decrease of the antioxidants CAT, GPx, SOD and GSH, along with an increase in MDA (*p* < 0.001) as compared with the other groups. The wood shavings-perlite and corncob groups had decreased MDA concentrations and significantly restored antioxidant levels. This was demonstrated by the increase in CAT content and GPx, SOD and GSH activities in comparison with the perlite and wood shavings groups. We did not observe any difference between sexes in any of the biochemical parameters except in SOD activity.

**TABLE 1:** Results of biochemical analysis performed on blood samples according to sex and cage-changing frequency in Sprague-Dawley rats.

Bedding material	Sex	Cage-changing frequency (per week)	CAT (kU/L) X	GPX (U/mL) X	MDA (mmol/L) X	SOD (U/mL) X	GSH (mmol/L) X
Wood shavings	Male	Once	208.684	0.258	16.600	15.251	2.592
		Twice	180.739	0.215	20.433	14.832	1.789
	Female	Once	216.401	0.252	16.033	14.804	2.084
		Twice	182.593	0.227	19.950	14.467	1.585
	Mean	-	197.104^a^	0.238^a^	18.254^c^	14.839^a^	2.012^a^
Wood shavings–perlite	Male	Once	192.927	0.211	18.600	14.835	1.506
		Twice	165.564	0.169	24.067	14.534	0.986
	Female	Once	181.679	0.197	18.183	14.953	2.146
		Twice	168.514	0.174	21.900	14.081	1.147
	Mean	-	177.171^bc^	0.188^b^	20.688^b^	14.601^a^	1.446^b^
Perlite	Male	Once	177.732	0.166	25.383	10.991	1.263
		Twice	153.301	0.157	30.900	9.430	0.800
	Female	Once	179.193	0.194	24.933	11.352	1.120
		Twice	167.274	0.121	30.520	7.840	0.483
	Mean	-	169.375^c^	0.159^c^	27.934^a^	9.903^b^	0.916^c^
Corncob	Male	Once	201.860	0.251	17.817	15.162	1.974
		Twice	161.206	0.214	23.383	14.982	1.446
	Female	Once	195.269	0.252	18.233	14.534	2.187
		Twice	184.402	0.238	22.790	13.582	1.427
	Mean	-	185.684^ab^	0.239^a^	20.555^b^	14.567^a^	1.758^ab^
Standard error mean	-	-	8.686	0.08	1.096	0.281	0.173
ANOVA	Bedding material	-	***	***	***	***	***
	Cage-changing frequency	-	***	***	***	***	***
	Sex	-	NS	NS	NS	***	NS

*Source*: Department Biochemistry, Faculty of Veterinary Medicine, Erzurum, Turkey

ANOVA, analysis of variance; CAT, Catalase; GPX, Glutathione peroxidase; MDA, Malondialdehyde; SOD, Superoxide dismutase; GSH, Glutathione.

X, values are mean.

*** Significant (*p* < 0.001); NS, Not Significant (*p* > 0.05);

abc significance of difference between same column (*p* < 0.05); one-way ANOVA and Tukey’s multiple comparison test; *n* = 96.

## Ethical considerations

The experiments were conducted according to the ethical norms approved by the Ethics Committee of Experimental Animal Teaching and Research Center (ATADEM-TR 2013/256).

## Discussion

The results demonstrate that bedding (wood shavings, wood shavings-perlite, perlite and corncobs) and bedding-change frequency had no significant effect (*p* > 0.05) on body weight in Sprague-Dawley rats. However, previous studies have reported that bedding material can alter the body weight (Anand Babu & Prasad 2014; Burn et al. 2006). Similarly, in this study, although bedding material did not significantly affect (*p* > 0.05) body weight, body mass increase was highest in the rats with the wood shavings-perlite combination.

Male rats are more likely than female rats to gain body weight (Peace et al. 2001). In this study, bedding changes once or twice a week did not significantly affect body weight (*p* > 0.05). This is probably because of the effect of less frequent bedding changes on animal well-being and healthiness. For this reason, weekly cage cleaning might be preferable because of cost and resource considerations. It has been stated that a twice-weekly cleaning of bedding for breeding rats results in more cannibalism of pups than weekly or biweekly cleaning (Burn et al. 2006). Moreover, cage changes have effects on health, behaviour and stress that stimulate fighting among rats (Burn & Mason 2008; Van Loo et al. 2004). For these reasons, bedding is commonly replaced about once a week (Bind et al. 2013; Ferrecchia et al. 2014). In our study, a once-per-week cage change interval, compared with a twice-per-week cage change, would expose rats to fewer stressful events; husbandry staff would be exposed less often to bedding materials; and facilities would benefit from reduced costs.

In this study, total bacterial count was lowest in the perlite and wood shavings-perlite groups. This is probably because of the bacteriocidal expanded perlite (Ibarguren et al. 2010). Although a previous study reported that total bacteria count was higher in sawdust than in corncob bedding (Anand Babu & Prasad 2014), our results showed that wood shavings resulted in less bacterial growth than corncob bedding. This discrepancy may be related to the specific characteristics of corncob bedding, which hosts various types of bacteria (Domer et al. 2012). Some bedding materials may not provide sufficient urine absorption and bacterial regulation to minimise ammonia production (Ferrecchia et al. 2014).

In this investigation, the wood shavings-perlite combination produced fewer degenerative and inflammatory skin reactions than perlite alone. There are no data in the literature about the connection between dermatitis and the use of corncob and wood shavings bedding materials for laboratory animals. However, some researchers have shown that using corncobs and sawdust as bedding materials for broilers is associated with dermatitis and that corncob has a more drastic effect on skin than sawdust (Xavier et al. 2010). Furthermore, in this study, changing bedding once weekly for both the wood shavings and corncob bedding groups resulted in mild degenerative and inflammatory reactions, while changing bedding twice weekly caused more severe skin reactions. The findings of the current study also correspond to a previous report, which investigated the inflammatory and irritant effects of perlite on skin in male Sprague-Dawley rats (Dracheva et al. 2011).

The term *antioxidant* refers to any molecule capable of stabilising or deactivating free radicals before they attack cells. Humans have developed highly complex antioxidant systems (enzymatic and non-enzymatic) that work interdependently and in combination with each other to protect body cells and organ systems against free radical damage. The body protects itself from the potential damage posed by ROS by utilising antioxidant enzymes and non-antioxidant enzymes such as CAT, GPx, SOD and GSH (Sisein 2014).

Oxidative stress is defined as an imbalance between production and regulation of ROS. ROS, mainly free radicals, are directly involved in oxidative damage to cellular macromolecules such as lipids, proteins and nucleic acids in tissues. In particular, biological membranes rich in unsaturated fatty acids are cellular structures exposed to free radical attack. MDA is the breakdown product of the major chain reactions leading to the oxidation of polyunsaturated fatty acids and thus causing oxidative stress. There are also antioxidant defence systems against different oxidants in the organism. These systems such as antioxidant vitamins, CAT, GPx, SOD and GSH protect the cells against lipid peroxidation. The above process gives rise to free radical insult termed lipid peroxidation or oxidative degradation of polyunsaturated fatty acids. Hence, a quantifiable end-product of lipid peroxidation is MDA, which is formed by radical cleavage reactions of polyenoic acids (Nwaigwe et al. 2010; Yang et al. 2010).

In the enzymatic antioxidant defence system, SOD is a copper–zinc-containing enzyme that converts superoxide into hydrogen peroxide and oxygen (Dubey, Raina & Khan 2012). Hydrogen peroxide is then either decomposed by catalase or reduced by a GSH dependent mechanism catalysed by GPx. Plasma catalase, GPx, SOD and GSH are antioxidant enzymes that deactivate the harmful effects of free radicals and prevent oxidative stress (Halliwell 2012). The function of these enzymes is protection from the potential damage of free oxygen radicals (Sisein 2014). There are no published data on the oxidative stress of perlite, wood shavings-perlite and corncobs as bedding materials for rats. Our findings have demonstrated that the use of perlite as a bedding material caused oxidative stress in rats, but the use of wood shavings and the wood shavings-perlite combination as bedding material, substantially reduced oxidative stress.

## Conclusion

In this study, perlite as the only bedding material had no effect on body weight, but it resulted in less microbial activity compared with other bedding materials. However, using perlite alone had negative effects on the skin, moisture percentage and stress parameters. Because the wood shavings-perlite combination appeared superior to perlite alone, it is important to determine an appropriate ratio of perlite and other bedding material that would improve animal health and welfare. Changing a wood shavings-perlite combination bedding on a weekly basis resulted in a lower moisture content and reduced bacterial activity in the bedding as well as affecting stress parameters positively in rats. The addition of perlite to bedding material should, therefore, be considered in an attempt to improve the quality of bedding material and the well-being of laboratory rats.
